# Consumption of OLL1073R-1 yogurt improves psychological quality of life in women healthcare workers: secondary analysis of a randomized controlled trial

**DOI:** 10.1186/s12876-021-01793-7

**Published:** 2021-05-24

**Authors:** Tetsu Kinoshita, Koutatsu Maruyama, Keiko Suyama, Mariko Nishijima, Kimiko Akamatsu, Akiko Jogamoto, Kikumi Katakami, Isao Saito

**Affiliations:** 1 Special Course of Food and Health Science, Department of Bioscience Graduate School of Agriculture, Ehime University, Matsuyama, Ehime Japan; 2 Institute of Community Life Sciences Co., Ltd., Matsuyama, Ehime Japan; 3 Department of Community Health System Nursing, Ehime University Graduate School of Medicine, Toon, Ehime Japan; 4 Department of Fundamental and Clinical Nursing, Ehime University Graduate School of Medicine, Toon, Ehime Japan; 5 Department of Public Health and Epidemiology, Faculty of Medicine, Oita University, Yufu, Japan

**Keywords:** Yogurt, *Lactobacillus delbrueckii* ssp. *bulgaricus* OLL1073R-1, Woman’s health, Quality of sleep, Psychological quality of life

## Abstract

**Background:**

We conducted a randomized controlled trial to investigate the effects of consumption of yogurt fermented with *Lactobacillus delbrueckii* ssp. *bulgaricus* OLL1073R-1 in women healthcare workers. In a previous study we used these data to investigate hypothesized preventive effects against flu, however any effects on improving mental quality of life were not analyzed at that time. In the present study, we focus on that aspect.

**Methods:**

The participants (961 women; mainly nurses, aged 20–71 years) were randomly allocated to either the yogurt group (n = 479) or the control group (n = 482). Participants in the yogurt group drank 112 mL of OLL1073R-1 yogurt for 16 weeks, while those in the control group did not consume any yogurt. All participants were prohibited from consuming other yogurt or fermented dairy products during the study period. The participants answered the Pittsburgh Sleep Quality Index (PSQI), Short Form-8 Health Survey (SF-8), and Gastrointestinal Symptom Rating Scale (GSRS) questionnaires at baseline and after 16 weeks.

**Results:**

The PSQI score showed significant improvement after the intake of yogurt (*p* < 0.01). SF-8 results showed significant intervention effects in the General Health and Vitality scores (*p* = 0.02 and *p* = 0.01, respectively). In other subscales of SF-8, we did not observe significant effects of the yogurt. In the GSRS, daily intake of yogurt exerted a preventive effect on constipation (*p* = 0.03).

**Conclusions:**

Consumption of yogurt fermented with *Lactobacillus delbrueckii* ssp. *bulgaricus* OLL1073R-1 enhances subjective psychological quality of life by improving quality of sleep and gastrointestinal condition among women healthcare workers.

## Background

It has been established that yogurt, a fermented dairy product, has beneficial effects on certain gastrointestinal conditions including lactose intolerance, constipation, inflammatory bowel diseases, and *Helicobacter pylori* infection [1, 2]. Recent studies have investigated the role of yogurt in enhancing human immune function by changing the balance of the intestinal microbiota and stimulating the intestinal immune system via lactic acid-producing bacteria or substances produced by the bacteria [3, 4]. Furthermore, some studies have attempted to demonstrate the beneficial effects of probiotics on mental health, i.e., ameliorating depression, anxiety, fatigue, and sleep disturbance [5–11]. The reported results have however been inconsistent. Psychological disorders impair the social functions of individuals, reduce the production efficiency of workers, and cause comorbidity with physical disorders [12]. Therefore, the beneficial effects of probiotics with respect to improving mental health should be further investigated to reduce the burden of psychological disorders among the working population.

A previous study showed that yogurt fermented with *Lactobacillus delbrueckii* ssp. *bulgaricus* OLL1073R-1 (OLL1073R-1), which produces a large amount of exopolysaccharide (EPS), reduced the risk of catching a cold in elderly subjects. Moreover, improvements in quality of life (QOL) in areas such as “Lacks general motivation”, “Irritation”, “Stress,” and “Easily fatigued” were also observed in the study [13]. However, previous studies have not comprehensively evaluated the effect of yogurt on QOL-related overall health indices. Based on the previous results [13] and expected benefits of probiotics [1, 2], we hypothesized that consumption of OLL1073R-1 yogurt may exert beneficial effects on improving psychological QOL and QOL-related overall health among other populations.

We primarily conducted this randomized controlled trial to evaluate the effects of OLL1073R-1 yogurt on prevention of flu among women healthcare workers [14]. In the present paper, we as secondary analysis evaluate the effects on improving psychological QOL and QOL-related overall health. Women healthcare workers are constantly exposed to the risk of infection because they are in prolonged contact with patients suffering from infectious diseases. Their level of psychological QOL also may be lower than that associated with other occupations due to the irregular nature of shift work [15]. Many working women are also busy with childcare and housework, and thus especially in need of health support. For those reasons, we selected women healthcare workers as the participants in this trial.

## Results

Table 1 shows the baseline characteristics of the participants. At baseline, there was no significant difference identified between the two groups for any of the indices.

**Table 1:** Baseline characteristics of participants

	Yogurt group (n = 479)	Control group (n = 482)	*p* value
Age (years)	39.3 (11.5)	39.4 (11.4)	0.91*
BMI (kg/m^2^)	22.0 (3.5)	21.8 (3.5)	0.42*
*Occupation*			0.62**
Nurse	310 (64.7%)	312 (64.7%)	
Care worker	43 (9.0%)	33 (6.9%)	
Physical therapist/occupational therapist	36 (7.5%)	40 (8.3%)	
Others	90 (18.8%)	97 (20.1%)	
Irregular shift workers	220 (46.0%)	228 (47.3%)	0.67**
Living by herself	118 (24.6%)	120 (24.9%)	0.93**
Current smokers	47 (9.8%)	43 (8.9%)	0.64**

Values are expressed as the mean. Standard deviation or percentage is indicated in parentheses

*Wilcoxon's rank-sum test

**Chi-squared test

Table 2 shows the scores of PSQI, SF-8, and GSRS at baseline and after 16 weeks in each group. Mean PSQI scores at baseline and after 16 weeks were 5.50 and 5.03 in the yogurt group, and 5.33 and 5.22 in the control group, respectively. The observed intervention effect was statistically highly significant (*p* < 0.01). For SF-8, the intervention effects were significant in the GH and VT scores (*p* = 0.02 and *p* = 0.01, respectively). The mean GH scores at baseline and after 16 weeks were 49.2 and 50.1 in the yogurt group, and 49.1 and 48.9 in the control group, respectively. The mean VT scores at baseline and after 16 weeks were 49.6 and 50.5 in the yogurt group, and 49.4 and 49.2 in the control group, respectively. We did not observe significant effects in other subscales, physical component summary, and MCS. The intervention effect in the GSRS score reached statistical significance for constipation (*p* = 0.03). Geometric mean constipation scores at baseline and after 16 weeks were 1.74 and 1.72 in the yoghurt group, and 1.76 and 1.84 in the control group, respectively. No statistically significant intervention effects were observed in the other subscales and total score.

**Table 2:** Changes in the measurements during the trial period

	Yogurt group (n = 479)		Control group (n = 482)		*p* value*
	Baseline	16 weeks	Baseline	16 weeks	
PSQI score	5.50 (2.71)	5.03 (2.68)	5.33 (2.60)	5.22 (2.68)	0.007
*SF-8 score*					
PF (physical functioning)	50.6 (4.5)	51.0 (4.9)	50.2 (5.1)	50.2 (5.0)	0.23
RP (role physical)	50.1 (5.1)	50.4 (4.9)	49.8 (5.2)	49.8 (5.4)	0.58
BP (bodily pain)	49.3 (8.5)	50.7 (8.3)	49.6 (8.5)	50.3 (8.4)	0.18
GH (general health)	49.2 (6.2)	50.1 (6.5)	49.1 (6.5)	48.9 (6.7)	0.02
VT (vitality)	49.6 (5.7)	50.5 (6.2)	49.4 (6.0)	49.2 (6.1)	0.01
SF (social functioning)	48.6 (7.2)	49.4 (6.9)	48.9 (7.3)	48.7 (7.5)	0.06
RE (role emotional)	48.7 (5.9)	49.4 (5.6)	49.1 (5.6)	49.2 (6.0)	0.17
MH (mental health)	47.0 (6.9)	47.9 (6.7)	47.2 (6.5)	47.7 (7.1)	0.27
PCS (physical component summary)	49.8 (5.8)	50.4 (5.7)	49.5 (5.9)	49.5 (6.1)	0.15
MCS (mental component summary)	46.6 (6.9)	47.4 (6.7)	47.0 (6.5)	47.1 (7.2)	0.12
*GSRS*					
Total^†^	1.58 (0.54)	1.59 (0.56)	1.58 (0.58)	1.60 (0.54)	0.70
Reflux of acid^†^	1.23 (0.85, 1.79)	1.24 (0.85, 1.81)	1.23 (0.84, 1.78)	1.25 (0.86, 1.82)	0.54
Gastric pain^†^	1.33 (0.89, 1.99)	1.33 (0.90, 1.95)	1.30 (0.88, 1.92)	1.32 (0.91, 1.93)	0.55
Indigestion^†^	1.48 (1.02, 2.16)	1.47 (1.00, 2.16)	1.50 (1.03, 2.18)	1.46 (1.01, 2.12)	0.52
Diarrhea^†^	1.32 (0.88, 1.99)	1.37 (0.92, 2.04)	1.29 (0.89, 1.87)	1.31 (0.90, 1.91)	0.50
Constipation^†^	1.74 (1.09, 2.80)	1.72 (1.04, 2.83)	1.76 (1.06, 2.93)	1.84 (1.09, 3.11)	0.03

Values are expressed as mean. Standard deviation (− 1SD, + 1SD) is indicated in parentheses

PSQI, Pittsburgh Sleep Quality Index; GSRS, Gastrointestinal Symptom Rating Scale

*Two-way analysis of variance with repeated measures

^†^Geometric mean values

Table 3 shows the correlation matrix between the changes in PSQI, GSRS, and QOL scores in the yogurt group. In the VT and MCS scores, a weak inverse correlation with PSQI was demonstrated (r = − 0.26, − 0.22, respectively). No correlation between the changes in PSQI and GSRS scores was observed (r = 0.08).

**Table 3:** Correlation matrix between the changes in PSQI, GSRS, and QOL scores in the yogurt group

	PSQI		GSRS		SF-8 sub-categories						SF-8 summary scores			
					GH		VT		SF		PCS		MCS	
	r*	*p*	r*	*p*	r*	*p*	r*	*p*	r*	*p*	r*	*p*	r*	*p*
PSQI	1.00	–												
GSRS	0.08	0.094	1.00	–										
GH	− 0.16	< 0.001	− 0.12	0.009	1.00	–								
VT	− 0.26	< 0.001	− 0.12	0.007	0.41	< 0.001	1.00	–						
SF	− 0.08	0.072	− 0.03	0.50	0.26	< 0.001	0.36	< 0.001	1.00	–				
PCS	− 0.07	0.12	− 0.18	< 0.001	0.45	< 0.001	0.36	< 0.001	0.14	0.003	1.00	–		
MCS	− 0.22	< 0.001	− 0.12	0.010	0.23	< 0.001	0.36	< 0.001	0.58	< 0.001	− 0.31	< 0.001	1.00	–

*Spearman's rank correlation coefficient

PSQI, Pittsburgh Sleep Quality Index; GSRS, Gastrointestinal Symptom Rating Scale; GH, general health; VT, vitality; SF, social functioning; PCS, physical component summary; MCS, mental component summary

There were no serious side effects caused by the intake of yogurt [14].

## Discussion

The primary outcome of the trial was to evaluate the preventive effects of yogurt fermented with *L. bulgaricus* OLL1073R-1 against influenza [14]. The present paper covers secondary outcomes of the same trial, not covered in the previous report: namely, sleep quality, subjective QOL, and gastrointestinal condition among women healthcare workers. Significant improvements in PSQI score, psychological QOL scores of the SF-8, and constipation score of the GSRS associated with yogurt intake were demonstrated.

A novel finding of the present study was improvement in the quality of sleep by the consumption of OLL1073R-1 yogurt. This effect may have significantly improved the GH and VT scores of the SF-8. As shown in Table 3, significant correlations were observed between the PSQI score and VT score. Therefore, we suspect that the observed increases in the GH and VT scores reflected reduced fatigue, itself attributable to improved sleep quality. A recent, randomized, placebo-controlled trial demonstrated the anti-fatigue effect of OLL1073R-1 yogurt among healthy male volunteers suffering from summer heat fatigue [16]. With respect to the GSRS, the intervention effect on the constipation score was statistically significant. This result suggests that constipation was prevented rather than exacerbated by the daily intake of yogurt (constipation score: 1.74–1.72). In the control group, constipation worsened due to the restrictions on yogurt intake (1.76–1.84). The effect of probiotics on improving dysbiosis has been documented in several previous clinical trials [17–20]. Benefits of probiotics have also been reported in the field of women-specific health (e.g. urology and gynecology) [21, 22].

The mechanisms of the involvement of yogurt fermented with *L. bulgaricus* OLL1073R-1 in the quality of sleep and fatigue are not fully understood. However, the EPS produced by OLL1073R-1 is considered one of its active ingredients. Several studies reported that purified polysaccharides exert an anti-fatigue effect [23–29]. Mushroom polysaccharide, known for its antioxidant activity, free radical scavenging activity, immunomodulatory activity, and maintenance of normal liver function, is involved in the anti-fatigue effect [30]. OLL1073R-1 EPS may exhibit anti-fatigue effects via similar mechanisms. In addition, it contains immunostimulatory phosphopolysaccharides [31] and possesses antioxidant activities, which have also been reported in EPS produced from another lactic acid bacterium [32–35].

The strength of the present study lies in the use of a large-scale, randomized, controlled design. A previous study which evaluated the anti-fatigue effect of the yogurt was small-scale [16]. The present results can provide new evidence in the field of nutritional psychology. Another strength is that this large-scale study was performed under stringently controlled conditions (i.e., participants did not consume any other fermented dairy products during the 16-week trial). Despite this restriction, the withdrawal rate was < 3% (n = 25), and > 900 participants completed the trial.

This study had several limitations. Firstly, participants were not blinded, which means that no placebo foods were prepared for the control group. Since it was impossible to prepare a placebo yogurt with similar characteristics (i.e., appearance, taste, and flavor) and deliver the blinded yogurts (active or placebo) to each participant every week, we employed an open-label design. As a result, some participants in the control group who were eager to be in the yogurt group might have experienced some stress due to the complete yogurt restriction. On the other hand, the yogurt group participants would have been motivated to complete the trial. Consequently, the differences in the mood or stress levels between the groups might have affected the results. Secondly, since participants could not consume any fermented dairy products except for the test food (for the yogurt group) according to the rules of the study, a certain amount of selection bias might have been generated. To eliminate this effect, it may in future trials be desirable to select participants who do not have a habit of consuming probiotics.

## Conclusions

The present study involving women healthcare workers showed positive effects of OLL1073R-1 yogurt intake on improving quality of sleep, fatigue, sense of general health, and constipation. Our findings provide new insights into the field of nutritional psychology and contribute to a better comprehension for the benefits of probiotics in our society. Consumption of probiotics, including OLL1073R-1 yogurt, should support many working women in their immunological and psychological health. Further clinical trials employing appropriate sample sizes and randomized controlled trial designs are needed to validate these effects of yogurt or fermented dairy products.

## Methods

### Study design and participants

The trial used to generate the data employed a randomized, controlled, and open-label design. The main objective was to examine the effects of OLL1073R-1 yogurt on the incidence of influenza among women healthcare workers [14]. This paper details the secondary, psychological health-related outcomes monitored in the same trial.

The main contents of methods of the present trial were explained in the previous report [14]. Women (aged ≥ 20 years) who were currently employed as medical or welfare-related professionals at medical institutions in Ehime prefecture and could understand the study purpose were selected, with written informed consent. The exclusion criteria were as follows: (1) pregnancy; (2) contracting influenza during the period from July 2016 to the date of providing written informed consent; (3) allergic responses to dairy products; (4) lactose intolerance; (5) instructed to restrict calorie intake by a physician; (6) history of diseases involving the immune system (e.g., rheumatism, cancer, thyroid disorder, systemic lupus erythematosus, myasthenia gravis, Graves’ disease, scleroderma); (7) participation in other clinical trials within the past 3 months; and (8) judged as unsuitable by the principal physician for other reasons. Of the 1026 women who agreed to participate in this study, 20 failed the exclusion criteria and 24 declined to participate after the agreement. Therefore, 982 women were examined at the screening session. By the day of the examination session, 20 further women failed the exclusion criteria and one declined participation. Consequently, 961 women (aged 20–71 years) were enrolled in the present study.

All participants were instructed not to consume any yogurt or fermented dairy foods from the day of agreement to that of random assignment. Participants were assigned to either the yogurt group (n = 479) or the control group (n = 482) through block randomization within three strata: institutions of employment, age, and having a plan of influenza vaccination or not.

**Fig. 1:**
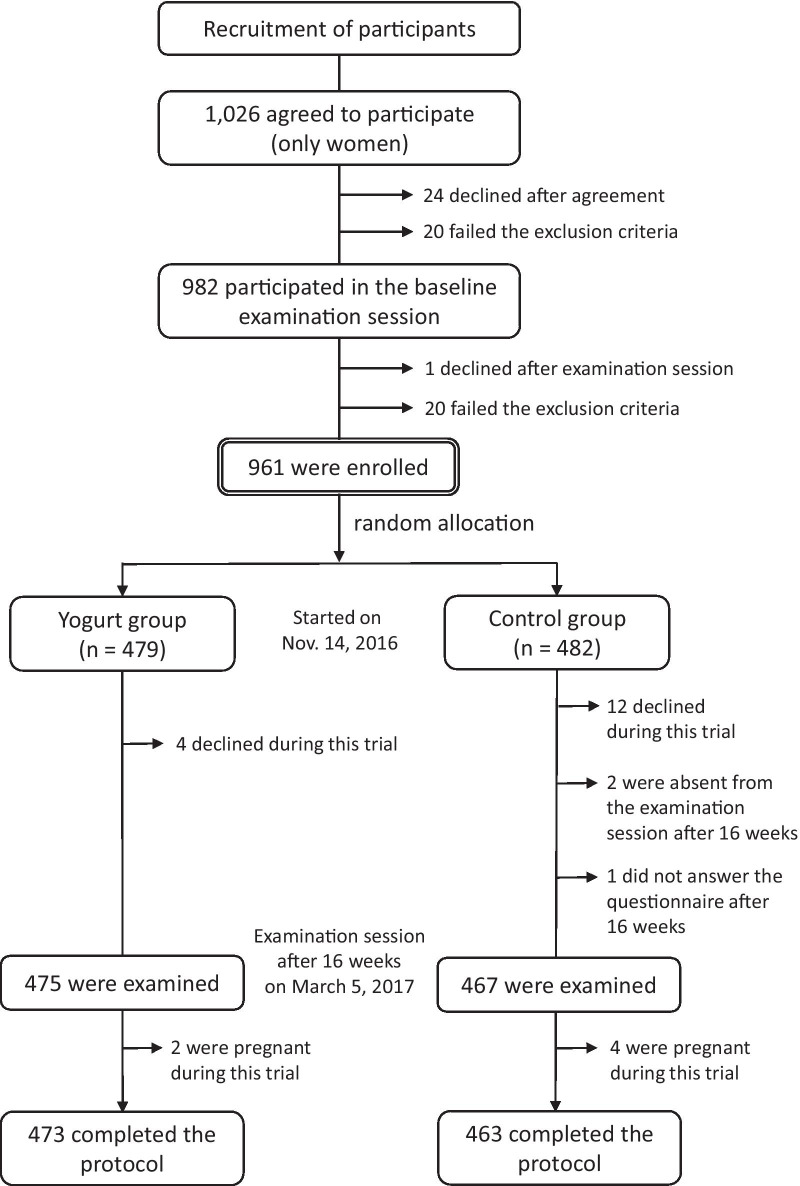
Sampling scheme throughout this study

Participants in the yogurt group consumed the test yogurt daily for 16 weeks (from November 14, 2016 to March 5, 2017), whereas those in the control group did not consume yogurt during this period. Furthermore, all participants were instructed not to consume any other yogurt or fermented dairy products throughout this trial. The participants answered self-administrated questionnaires at baseline and after 16 weeks. In addition, participants were provided with a “health notebook” to record changes in their lifestyles, intake of the test yogurt (for the yogurt group), and intake of any other yogurt or fermented dairy foods (for both groups) during the trial period. During the trial, four and 12 participants in the yogurt and control group declined participation, respectively. In the control group, two participants did not attend the examination session and one did not complete the self-administered questionnaires 16 weeks later. In addition, two and four participants in the yogurt group and control group, respectively, revealed that they were pregnant during the trial, thereby infringing the exclusion criteria. Figure 1 shows the sampling scheme throughout this study.

### Test yogurt

The test food was “Meiji Probio Yogurt R-1” drink type (Meiji Co., Ltd., Tokyo, Japan), which is currently available on the market. This yogurt is manufactured using two lactic acid bacterial species, namely *L. bulgaricus* OLL1073R-1 and a strain of *Streptococcus thermophilus*, originally isolated from traditional Bulgarian yogurt. One bottle contains 112 mL of drinkable yogurt and provides 76 kcals, 13.9 g carbohydrate, 0.67 g fat, 3.6 g protein, and ≥ 1.12 × 10^9^ CFU (colony forming units) of *L. bulgaricus* and *S. thermophilus*.

### Outcome measurement

The participants completed the self-administrated questionnaires discussed below at baseline and after 16 weeks.

### Quality of sleep

The Pittsburgh Sleep Quality Index (PSQI) was used as an index of subjective sleep condition [36], as it has been established that dairy products exert beneficial effects on relaxation and quality of sleep [37]. The questionnaire is self-administered, and the participants were asked to answer the questions according to their quality of sleep (i.e., onset, duration, efficiency, difficulty, medication, and daytime sleepiness) during the previous 1 month. Each answer is converted into the score and the total score reflects the comprehensive sleep condition. Higher scores represent worse condition.

### Subjective QOL

The eight-item Short Form Health Survey (SF-8) was used to evaluate subjective QOL [38]. The effect of OLL1073R-1 on improving mood status have been previously reported [13]. Therefore, we examined its role in improving psychiatric QOL. The SF-8 is a generic questionnaire widely used to compare the impact of different medical conditions, and as an outcome measure of different therapeutic interventions. The questionnaire is self-administered, and the participants were asked to complete eight questions according to their experiences during the previous 1 month. The questions were divided into eight subscales and two dimensions that described the overall health status. The eight subscales were physical functioning (PF), role physical (RP), bodily pain (BP), general health (GH), vitality (VT), social functioning (SF), role emotional (RE), and mental health (MH). The two dimensions were physical component summary (PCS) and mental component summary (MCS), which were calculated based on the scores of the eight subscales using specific standardized algorithms. The scores of each scale ranged from 0 to 100, with high scores representing better QOL.

### Gastrointestinal condition

The Gastrointestinal Symptom Rating Scale (GSRS) was used as an index of gastrointestinal condition [39, 40], as it has been established that probiotics improve gastrointestinal symptoms, such as constipation [2]. The GSRS consists of 15 questions; the participants answered all questions according to their gastrointestinal condition during the previous 1 week. We evaluated the total score and five subscale scores (reflux of acid, gastric pain, indigestion, diarrhea, and constipation). The scores of the total and each subscale ranged from 1 to 7, with higher scores representing worse condition.

### Statistical analysis

All statistical analyses were performed using SAS version 9.4 (SAS Institute Inc., Cary, NC, USA) based on intention-to-treat analysis. Differences in the means of the indices between the two groups at baseline and after 16 weeks were analyzed using an unpaired t-test. The effects of the intervention on the scores of the PSQI, SF-8, and GSRS were analyzed through two-way analysis of variance with repeated measures. Owing to the non-normal distribution of the GSRS score, geometric means were calculated and used for analysis. In case of participant withdrawal from the study, the values for PSQI, SF-8, and GSRS obtained at baseline were used as those after 16 weeks. In the yogurt group, the correlation between the changes in QOL scores and changes in PSQI and GSRS scores were analyzed using Spearman’s rank correlation analysis. For the incidence of adverse events in both groups, the rates were analyzed in 10 categories using the chi-squared test. In all analyses, *p* < 0.05 (two-sided) denoted statistical significance.

### Ethics statement

The present study was approved by the Institutional Review Board (IRB) of Ehime University Hospital and the IRB of the Meiji Corporation. Written informed consent was provided by all participants. The reporting of this trial follows the recommendations of the CONSORT (Consolidated Standards of Reporting Trials) 2010 statement [41]. This trial was registered at the University Medical Information Network Clinical Trial registry as UMIN-RCT Identifier UMIN000023932 (10/09/2016). In addition, the study was conducted in accordance with the protocol and Japanese ethical guidelines for medical and health research involving human subjects.

## Data Availability

The datasets generated during the current study and analyses are available from the corresponding author on reasonable request.
